# Association Between Pulmonary Tuberculosis and Bronchial Anthracosis

**DOI:** 10.7759/cureus.73499

**Published:** 2024-11-12

**Authors:** Khalid Ahmed, Kamran K Sumalani, Nousheen Akhter, Maqbool Ahmed, Abdul Baqi, Nadeem Rizvi

**Affiliations:** 1 Pulmonology, Fatima Jinnah General and Chest Hospital, Quetta, PAK; 2 Pulmonology, Jinnah Postgraduate Medical Centre, Karachi, PAK; 3 Pulmonology, Bahria University Health Sciences Campus, Karachi, PAK; 4 Pulmonology, Shaikh Khalifa Bin Zayed Al-Nahyan Medical Complex, Quetta, PAK

**Keywords:** anthracosis, biomass fuel, bronchoscopy, tuberculosis, xpert mtb/rif assay

## Abstract

Introduction

Anthracosis is black discoloration of the bronchi, which can sometimes cause anthracofibrosis. Usually, exposure to biomass fuel, air pollution, or smoke at the workplace causes it. The objective of the current study was to determine the association between tuberculosis and anthracosis.

Methods

Patients with chronic dyspnea, dry cough, and infiltrates or mass lesions on chest X-rays underwent bronchoscopic lavage and biopsy where needed. Patients were divided into two groups on the basis of anthracosis on bronchoscopy. Diagnosis of tuberculosis was made on bronchial wash acid-fast bacilli smear, mycobacterial culture, or Xpert Mycobacterium tuberculosis (MTB) rifampicin (RIF) assay.

Results

Tuberculosis was diagnosed by bronchoscopy in 68/173 (39.30%) patients with anthracosis which was significantly higher (p-value 0.020) than the control group (43/159, 27.04%). Most of the cases (54/68, 79.41%) had positive Xpert assay. The male-to-female ratio was 3:1, and all the females with anthracosis were homemakers and used biomass fuel.

Conclusion

Patients with exposure to smoke are prone to develop anthracosis. The presence of anthracosis is associated with the development of tuberculosis. Appropriate investigations for tuberculosis must be done in subjects undergoing bronchoscopy who are found to have anthracosis.

Expanding understanding among the masses regarding the hazards of using biomass fuel in closed spaces can be a crucial measure toward diminishing the chance of developing anthracosis and the concomitant risk of tuberculosis.

## Introduction

Anthracosis is defined as a black discoloration of the bronchi, and it can sometimes cause bronchial narrowing, also known as anthracofibrosis [1]. It is an ancient disease, and the record dates back to 18th-century mummies [2,3]. The exact prevalence of the disease is not known because its confirmatory diagnosis requires bronchoscopy, which is not doable to screen the general population. Available data has shown the frequency of simple anthracosis to be 3.4-21% [4,5] and that of anthracofibrosis to be approximately 10.09% [6].

The exact etiology of anthracosis is unknown. Possible causes include coal dust, biomass fuel, and tuberculosis [7]. According to Global Health Observatory Data, 46% of the world’s population and 63.4% of Pakistan’s population live in rural areas. Indoor air pollution from burning biomass fuel is the most important environmental cause of mortality and morbidity [8]. It is considered one of the most common causes of anthracosis in people living in rural areas and cold mountain areas.

The clinical presentation of anthracosis is similar to that of chronic obstructive pulmonary disease. Earlier literature shows an association between tuberculosis (TB) and anthracosis, and patients with worsening clinical symptoms required an evaluation to rule out tuberculosis, but later it could not be proven [1,9]. There is growing interest among clinicians to ascertain if ‘anthraco-tuberculosis’ is an emerging medical condition in high TB burden areas.

The inhalation hypothesis does not explain localized anthracosis. The assumption is that pulmonary tuberculosis may be a cause of localized anthracosis and anthracosis in patients without a history of exposure to smoke [10]. On the contrary, studies about a century ago in coal mine workers have revealed protective and bordering effects of anthracotic lesions for tuberculosis [11].

Tuberculosis is endemic in Pakistan. Nearly two-thirds of the Pakistani population lives in rural areas and uses biomass fuel for cooking or keeping the house warm; which causes anthracosis. There is no large-scale study on anthracosis and tuberculosis from Pakistan. Timely recognition of tuberculosis is important to reduce morbidity and mortality in people with anthracosis.

The current study aimed to determine the association of active tuberculosis in patients with anthracosis.

## Materials and methods

This was a case-control study conducted in the Department of Pulmonology, Jinnah Postgraduate Medical Center, Karachi, from March 2016 until March 2018.

A total of 332 patients with undiagnosed chronic dyspnea and dry cough, having parenchymal infiltrates, atelectasis, or mass lesions on chest X-rays, were included in the study (173 cases and 159 controls). Demographic details including age, gender, smoking status, occupation, duration of exposure to biomass or coal dust, and comorbid illnesses were recorded. High-resolution computed tomography (HRCT) scans and flexible bronchoscopy were done for all patients, and findings were recorded. Samples of bronchial lavage fluid were sent for acid-fast bacilli (AFB) smear, Xpert Mycobacterium tuberculosis (MTB) rifampicin (RIF) assay, AFB culture, fungal culture, and cytology. An accessible bronchoscopic biopsy of the mass lesion was done and sent for histopathology. Patients were divided into two groups on the basis of appearances on bronchoscopy. Patients with anthracosis were labeled as cases, and those with no anthracosis as controls.

Tuberculosis was diagnosed if bronchial lavage fluid came out positive for AFB smear, Xpert assay, or AFB culture. Non-smoker patients aged more than 40 years of either gender with undiagnosed chronic dyspnea and/or dry cough, exposure to burning biomass fuel or coal dust for more than 10 years, and having parenchymal infiltrates, atelectasis, or mass lesion on chest X-rays were included in the study. Patients not meeting these criteria or who had a history of tuberculosis were excluded.

Written informed consent was taken from all study participants. The study was approved by the hospital's ethical review committee (letter number F.2-81/2015-GEN/9820/JPMC, dated 13/07/2015).

Statistical analysis was done using IBM SPSS Statistics for Windows, Version 20 (Released 2011; IBM Corp., Armonk, New York, United States). A P-value < 0.05 was taken as statistically significant.

## Results

The mean age of the anthracosis group was 60.65 years (±7.33), and the female-to-male ratio was 3:1. All of the females with anthracosis were homemakers using biomass fuel in stoves. The clinico-demographic profile of cases and controls is given in Table 1.

**Table 1:** Clinico-demographic profile of patients.

	Cases	Controls	p-value
Age in years (mean ± standard deviation)	60.65 ± 7.33	58.59 ± 7.29	0.02
Gender (Male/Female)	33/99	20/93	0.21
Occupation			0.18
Homemaker	99	93	
Miner	9	3	
Baker	15	14	
Other	9	3	
Duration of exposure			
20 – 30 years	15	38	<0.001
31 – 40 years	56	32	
> 40 years	61	43	
Comorbid illness			
Diabetes mellitus	51	24	0.00
Hypertension	50	33	0.17
Ischemic heart disease	26	19	0.62
Chronic liver disease	13	9	0.65
Chronic kidney disease	16	6	0.07
Malignancy	0	6	0.00

HRCT chest showed findings of pulmonary infiltrates (47, 14.15%), consolidation (50, 15.06%), ground glass opacity (21, 6.32%), bronchiectasis (11, 3.31%), nodules (20, 76.02%), honeycombing (17, 5.12%), cavity (7, 2.10%), calcified lymph nodes (6, 1.80%), atelectasis (86, 25.90%), mass (59, 1.77%), and fibrosis (8, 2.40%). Tuberculosis was diagnosed by bronchoscopy in 68/173 (39.30%) patients with anthracosis which was significantly higher (p-value 0.020) than the control group (43/159, 27.04%). Most of the cases (54/68, 79.41%) had positive Xpert assay. The results of the bronchoscopic specimens for tuberculosis are shown in Table 2.

**Table 2:** Results of bronchoscopic investigations in anthracotic patients diagnosed with tuberculosis. MTB: Mycobacterium tuberculosis; RIF: rifampicin

Investigation	Frequency	Percentage
Bronchial lavage acid-fast bacilli smear	20	29.41
Bronchial lavage Xpert MTB/RIF assay	54	79.41
Bronchial lavage acid fast bacilli culture	68	100
Bronchoscopic biopsy	3	4.41

## Discussion

Anthracosis is said to be a disease of the elderly. There is a 1.07%-increased risk of developing anthracosis with each year's increase in age. In our study, the mean age was also higher, 60.65 ± 7.33 years. The current study demonstrates a higher prevalence of tuberculosis in anthracotic subjects than the control group (68/173 versus 43/159, OR 1.74). The prevalence of pulmonary tuberculosis is reported between 6.9% and 57.8% in previously published literature [9,12-14].

Tuberculosis is an ancient disease, but it remains a critical health concern around the globe. Its diagnosis is difficult because of the uncertain nature of tuberculosis [15]. It causes varied respiratory symptoms, like endobronchial tuberculosis that may present with wheezing and remains misdiagnosed as bronchial asthma for long. Tuberculosis is diagnosed by bacteriological tests, and they give confirmatory evidence of TB. In previous literature [9], tuberculosis was diagnosed by AFB smear in 96% of cases. The most sensitive test for tuberculosis is reported to be mycobacterial culture in another study [10]. In some studies, histopathology remained the mainstay of diagnostic investigation for the diagnosis of tuberculosis [16,17]. In our study, endobronchial wash AFB culture was positive in all anthracotic patients with tuberculosis, and MTB Xpert was positive in 79.41%, giving the diagnosis in a day.

The burning of biomass fuel for cooking is an established causative factor for endobronchial anthracosis. A study reported that the Indian subcontinent has a higher rate of development of anthracofibrosis (50%) than other Asian countries (3.7%) [6]. One speculation regarding the occurrence of TB in anthracotic patients is that inhaled silica particles may curb the function of alveolar macrophages, thereby decreasing the body’s defense against Mycobacterium tuberculosis [13]. Inhalation of dust, carbon, silica, etc. are among the predisposing factors for the development of anthracosis. This may cause generalized anthracosis. The etiology of localized anthracosis cannot be explained by these exposures. The assumption prevails that pulmonary tuberculosis may be the underlying cause of localized anthracosis and also for anthracosis in non-smoking subjects or those with no work-related exposures [1,18]. It is believed that tuberculous lymph nodes rupture into the endobronchial lumen, causing localized endobronchial narrowing and anthracosis [1].

The overall health status of the people is linked to tuberculosis. There is an inverse relationship between tuberculosis and good socioeconomic status and health. This will include smoking cessation, education regarding workplace exposures, and biomass fuel exposure, which have a causal link to anthracosis. A Korean study showed that the frequency of tuberculosis was 60% in anthracosis. However, recent studies reported it to be in the range of 20-34% [1,19,20]. In privileged countries, the frequency of tuberculosis is lower in anthracosis as overall health status is better. In a study done in England, only one patient had tuberculosis as a causative of anthracosis [1]. The incidence of tuberculosis in anthracosis is in the range of 20-30% during bronchoscopy, as quoted by various studies [9,10,16,17,19-21].

Tuberculosis must be kept in mind in anthracotic patients with unexplained dyspnea, wheezing, weight loss, and fever, especially those with localized anthracosis. Early treatment with antituberculous therapy helps improve symptoms and save the patient from developing the dreaded complication of endobronchial strictures.

There are certain limitations to the study. First, there may be selection bias. Secondly, this is a single-center study, so the results cannot be generalized to the whole population. Third, patients with prior histories of tuberculosis were excluded, but this exclusion was dependent on patients’ recall. TB is endemic in the region; it may happen that one has developed TB and healed on its own without the patient knowing. Fourth, latent TB was not taken into account. Further large-scale multi-center studies can be undertaken in the future on this subject.

## Conclusions

Patients with anthracosis have mild respiratory symptoms and few imaging findings. Anthracosis is diagnosed by a flexible bronchoscopy. Its association with TB is of great importance. Patients exposed to biomass fuel, especially, are prone to developing anthracosis, and the disease shall be looked for in these cases; if present, then keep a high suspicion for tuberculosis too. Appropriate investigations for tuberculosis must be done in subjects undergoing bronchoscopy who are found to have anthracosis.

Increasing public awareness of the risks associated with burning biomass fuel in enclosed areas can be a key step in reducing the incidence of anthracosis and the associated risk of tuberculosis.
